# Case Report: Keep your eyes open! Nystagmus guides atypical BPPV

**DOI:** 10.3389/fresc.2024.1384151

**Published:** 2024-03-28

**Authors:** Daniel Ludwig, Michael C. Schubert

**Affiliations:** ^1^ Department of Physical Medicine and Rehabilitation, Johns Hopkins University, Baltimore, MD, United States; ^2^ Laboratory of Vestibular NeuroAdaptation, Department of Otolaryngology-Head and Neck Surgery, Johns Hopkins University, Baltimore, MD, United States

**Keywords:** BPPV, positional vertigo, positional nystagmus, vestibular, atypical

## Abstract

The clinical diagnosis of benign paroxysmal positional vertigo (BPPV) is confirmed from observing the direction, intensity, and duration of nystagmus from unique head positions that advantage gravity to overcome the inertia of otoconia displaced inside the semicircular canals. This case series highlights BPPV with atypical nystagmus presentations relative to the head position. Clinicians should carefully observe symptoms and nystagmus presentations regardless of the testing position and utilize technology and rules of vestibular physiology to enhance their diagnostic acumen.

## Introduction

Variants of benign paroxysmal positional vertigo (BPPV) such as sitting up vertigo, short-arm posterior canal BPPV, and type II BPPV may present with atypical nystagmus patterns or even absent nystagmus that can be difficult for clinicians to manage and cause longer durations of morbidity (1, 2). This case series presents four cases of unique BPPV presentations that highlight the clinical means to appropriately identify the affected semicircular canal based on nystagmus patterns that include comparing the intensity of nystagmus in different head positions, changing gaze direction (eye in orbit) to accentuate vertical or torsional components, and identifying canal-specific nystagmus patterns independently of the positioning test being performed.

All patients underwent a clinical oculomotor exam (smooth pursuit, gaze stability, saccade) including fixation removed testing (VestibularFirst Broomall, PA), video head impulse testing (GN Otometrics, Denmark), and tests of labyrinthine integrity with fixation removed (tragal pressure, glottis closed Valsalva). These tests were all normal unless otherwise noted in their case.

### Case 1: excitatory nystagmus in bilateral Dix–Hallpike testing from unilateral posterior semicircular canal BPPV

A 42-year-old woman with a history of vitamin D deficiency, family history of migraine, and prior episodes of successfully treated BPPV presented for re-assessment after developing her typical vertigo symptoms when rolling to the left in bed overnight, getting out of bed in the morning, and laying supine during an exercise class.

Right and left supine roll test (SRT) were negative for nystagmus and vertigo. Right Dix–Hallpike test (DHT) showed upbeating and left torsional nystagmus after a 6 s latency that then was persistent beyond 40 s (Supplementary Video S1). The patient did not report vertigo in right DHT but felt her eyes “pulsating”—consistent with the observed nystagmus. In the left DHT, she developed an immediate onset of upbeat and left torsional nystagmus with a crescendo–decrescendo velocity pattern that was greater compared with the right side and accompanied with vertigo. Although the nystagmus persisted for more than 60 s (Supplementary Video S1), it did slow down and thus an initial treatment of a left Epley canalith repositioning maneuver (CRM) was applied. However, while in the third position of the CRM she developed a mild downbeating nystagmus with right torsion that was persistent without vertigo, suggesting cupulolithiasis of the left posterior semicircular canal. She had no nystagmus reversal upon return to sitting. Repeat testing and a second CRM produced similar results. Next, a Semont-plus maneuver was performed for the left posterior semicircular canal and she had a burst of excitatory nystagmus (upbeating with left torsion) in the initial left-sidelying position, which reversed to prolonged downbeat nystagmus in the nose down and right sidelying position that extinguished after 90 s.

The patient was scheduled for a follow-up session 5 days later but cancelled the appointment as she was no longer having symptoms, suggestive of successful treatment. She has not returned to the clinic.

#### Case pearls and possible mechanisms

**Figure 1:**
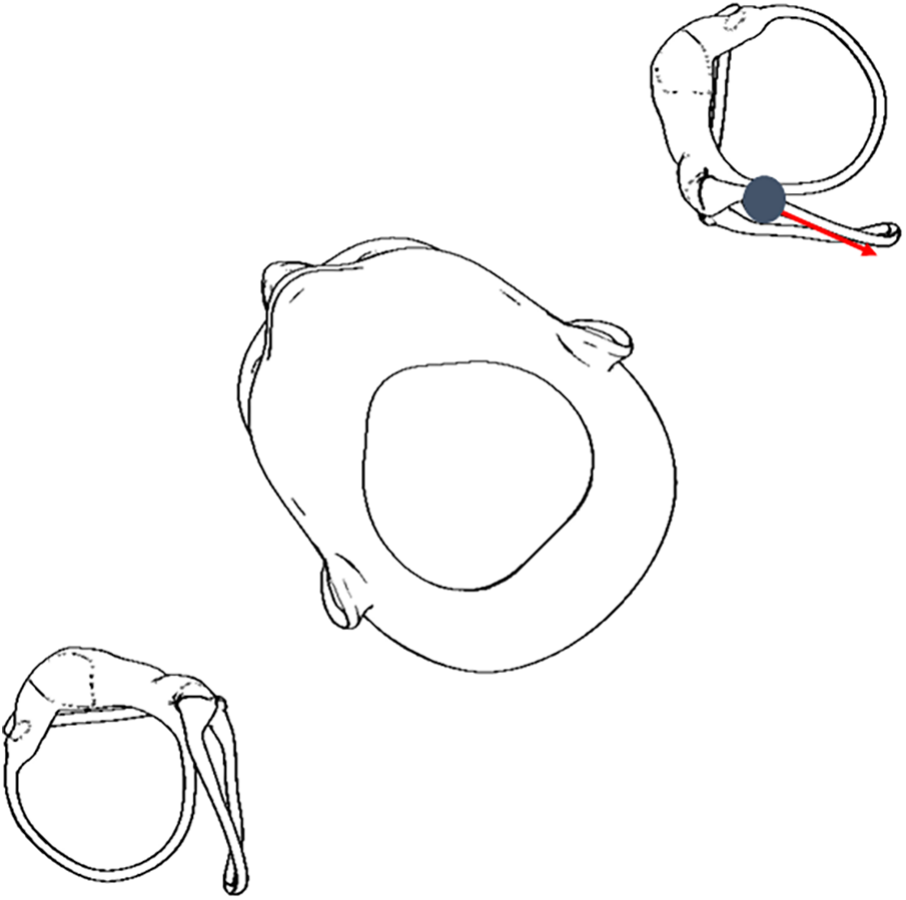
Otoconia displaced in the left posterior semicircular canal can move in an excitatory direction in a right DHT (5, 6), therefore, it is critical for clinicians to perform the DHT for both sides.

This patient's case highlights the importance of observing the direction of the torsional component of positional nystagmus independent of the semicircular canal being tested. The patient had upbeating and torsional nystagmus in both DHT, which may confuse clinicians to diagnose bilateral posterior canal BPPV. However, careful observation revealed the left torsional component was accentuated by having the patient change her gaze (3), similarly in both the right and left DHT, consistent with excitation of the left posterior semicircular canal. Another clinical pearl for this case is the observed intensity of nystagmus and vertigo. The velocity of an excitatory cupular deflection in the left DHT (and resultant vertigo) was greater than that from the right DHT, consistent with the DHT intent of positioning the affected posterior semicircular canal in the most gravity-dependent position that should enable a more robust nystagmus (4), and resulted in a greater intensity of vertigo. The patient had no vertigo during the right DHT. Figure 1 illustrates how the left posterior semicircular canal could be excited during a right DHT.

The probable cause for the observed nystagmus in this case is left posterior canal cupulolithiasis, with otoconia adherent to the cupula explaining the persistent nature of the observed symptoms and nystagmus in both DHT and the lack of responsiveness to the left Epley CRM. Adherent otoconia would also account for the persistent downbeat nystagmus observed in the third position of the left Epley CRM and final position of the Semont-plus maneuver (7). Further clinical reminders include identifying the latency and duration of nystagmus and the fatigability of nystagmus on repeated testing (8) and deciding on the appropriate treatment strategy independent of a patient's prior BPPV diagnosis and response to treatment.

In this case, both latency and duration of nystagmus as well as repeated testing of nystagmus fatigability indicated a cupulolithiasis-type BPPV, despite the crescendo–decrescendo pattern and prior diagnosis (8).

### Case 2: excitatory and inhibitory nystagmus from unilateral posterior semicircular canal BPPV

A 44-year-old woman without any relevant medical history presented for evaluation of a 1-month history of episodic vertigo. The initial symptoms occurred when getting up from lying on the couch and lasted about 30 s. She reported feeling “off” and having a mild gait instability during the first day. After the initial onset, she experienced short episodes of vertigo lasting 5–10 s rising from supine and occasionally when rolling in bed. She reported that the vertical head motion would make her dizzy. She denied headaches, migraine symptoms, and any personal or known family history of migraines.

Right and left SRT were completed with no nystagmus or vertigo. Right DHT revealed downbeat and right torsional nystagmus lasting longer than 60 s (Supplementary Video S2). The patient reported generalized dizziness but not vertigo. Upon returning to sit from the right DHT, she had no nystagmus or vertigo. Left DHT revealed a robust upbeat and left torsional nystagmus lasting about 20 s with a 3 s latency consistent with a left posterior semicircular canalithiasis (Supplementary Video S2). This nystagmus then transitioned to a slow velocity downbeat nystagmus without symptoms.

The patient was treated with an Epley CRM for left posterior semicircular canalithiasis. Following treatment, repeat left DHT demonstrated a slow velocity downbeating nystagmus without vertigo or dizziness. There was no reversal of nystagmus and no symptoms when returning to sit. Repeat right DHT was negative for both vertigo and nystagmus. The patient has not returned to the clinic but indicated she has no ongoing symptoms when consent was obtained.

#### Case pearls and possible mechanisms

**Figure 2:**
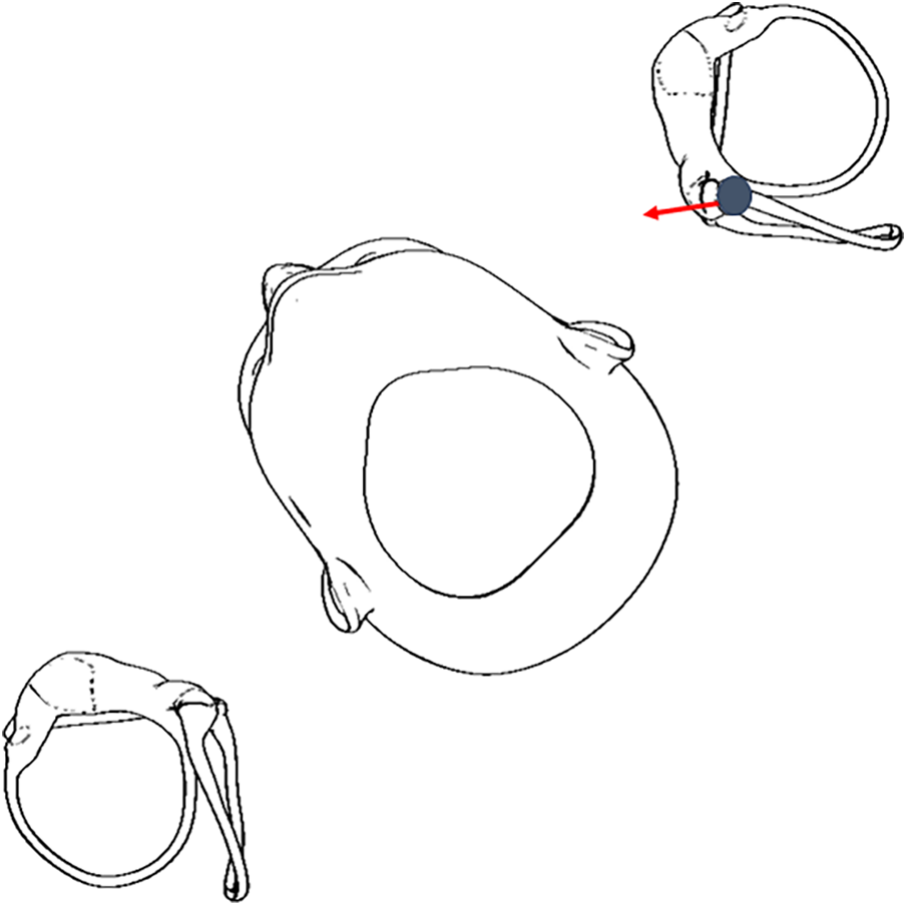
Otoconia displaced in the left posterior semicircular canal can move in an inhibitory direction in a right DHT (5, 6), therefore, it is critical for clinicians to perform the DHT for both sides.

The downbeat and right torsional nystagmus in the right DHT indicate two possibilities: (1) excitation of the right anterior semicircular canal or (2) inhibition of the left posterior semicircular canal (9). According to published diagnostic criteria for BPPV (10), anterior semicircular canalithiasis BPPV is rare and putatively can present with a predominantly vertical (downbeat) nystagmus. The addition of a straight head-hanging test position may have improved diagnostic efficiency as it has been shown to be more sensitive to true anterior semicircular canal canalithiasis (10, 11). Given the slower velocity downbeat with clearly observable right torsional nystagmus in the right DHT (consistent with an inhibitory response), coupled with a robust upbeat and left torsional nystagmus in left DHT—we reasoned the likely cause is the left posterior semicircular canal being inhibited and excited, respectively (Figure 2).

The absence of vertigo yet residual downbeat nystagmus during her repeat testing post CRM is not uncommon and has been reported to exist in 39% of patients being treated for posterior semicircular canal BPPV (12).

It is possible the otoconia from the left posterior semicircular canal were located within its short arm. Ping et al. (13) and later Ludwig and Schubert (1) reported excitatory nystagmus due to putative short-arm posterior canal BPPV. Residual otoconia in the short arm of the posterior canal following treatment could also account for the downbeat nystagmus observed in left DHT after treatment, although that is unlikely as she did not report vertigo.

### Case 3: excitatory nystagmus from unilateral posterior semicircular canal BPPV during the supine roll test

The patient was an 80-year-old man with a history of muscular dystrophy with incomplete penetrance, atrial fibrillation, non-ischemic cardiomyopathy after pacemaker placement, Type II diabetes mellitus, hypertension, and cervical spondylosis who presented as a return patient for positional vertigo. His initial visit to the clinic occurred 1 month prior and at that time he was treated successfully for left posterior semicircular canalithiasis using an Epley CRM. The patient's cervical range of motion was limited from thoracic kyphosis that required modification of the positional testing and CRMs.

The patient was initially brought from long sitting to supine with head flexed 30° in preparation for the SRT, and after a 3–4 s latency, developed an upbeat and left torsional nystagmus. Given the patient's history of left posterior semicircular canal BPPV, the clinician decided to forego SRT and immediately changed positioning to a left DHT, where the upbeat and left torsional nystagmus continued with a crescendo–decrescendo pattern for just over 60 s.

An Epley CRM was attempted without reproduction of symptoms or nystagmus throughout the maneuver. There was no reversal of nystagmus with return to sitting. A repeat left DHT demonstrated nystagmus consistent with the initial test, although this time lasting only about 40 s. A second Epley CRM was performed with similar results as the first. The clinician considered both nystagmus and the symptoms were refractory to an Epley CRM, and hence decided to retest using the left sidelying test (14), which offered the possibility to quickly treat using a Semont maneuver if positive.

Left sidelying test revealed the same pattern, duration, and intensity of nystagmus and symptoms as the second left DHT, and a Semont maneuver was completed. There were no symptoms or nystagmus after transitioning to the final position (nose down and right sidelying) of the Semont maneuver, nor when returning to sit. A final left sidelying test and Semont maneuver produced the same positive results as the previous test. Thus, the “sleep maneuver” for posterior canal BPPV (15) was prescribed to the patient for the home program, and he was scheduled to follow-up in the clinic in 2 days; however, he cancelled the appointment due to neck discomfort. He has not returned to the clinic.

#### Case pearls and possible mechanisms

This patient's case highlights the importance of appropriately identifying canal-specific nystagmus patterns independently from the positional test being performed. Early identification of mixed vertical and torsional nystagmus from horizontal nystagmus in this patient with recent history of posterior semicircular canal BPPV was helpful for the treating clinician to reduce the number of test positions in this elderly patient with musculoskeletal limitations.

One possible mechanism for this patient's presentation is a typical left posterior semicircular canalithiasis BPPV, which was refractory to treatment per his musculoskeletal range of motion limits. Other possible mechanisms include a short-arm posterior canal BPPV, but further positional testing on subsequent visits would be required to explore this.

### Case 4: excitatory and inhibitory nystagmus from multi-canal BPPV

The patient was a 74-year-old man with a history of hypertension, obstructive sleep apnea, and hyperlipidemia presenting for evaluation of 2-week onset of positional vertigo symptoms and gait unsteadiness. He reported positional vertigo symptoms when rolling over in bed and sitting up from supine. He also reported episodic gait instability where he had to hold onto furniture to walk and did not tolerate head movements or walking in low light environs. His oculomotor exam revealed a mild downbeat nystagmus after horizontal head shaking (fixation removed). See Table 1 for a summary of the results of positional testing and treatment across this patient's three visits.

**Table 1:** Summary of observed nystagmus patterns, durations, and symptoms including responses to attempted treatments across three treatment sessions for case four.

Positional test	Visit#	Observed nystagmus	Symptoms
Sit to supine	1	Mild downbeat	None
	2	Right beat	None
	3	Upbeat, left torsion ×10 s	None
Right SRT	1	Mild downbeat	None
	2	Initial—left beat (apogeotropic), persistent	Initial—none
		After Epley—left beat (apogeotropic), persistent, mild	After Epley—No vertigo, mild nausea
	3	Right beat, geotropic ×10 s	None
Left SRT	1	Right beat (apogeotropic), then mild downbeat, right torsion	None
	2	Initial—right beat (apogeotropic)—robust, ∼28 s transitions to upbeating w/left torsion ×17 s, then returns to right beat (persistent)	Initial—intense
		After Epley—right beat (apogeotropic), intense and persistent	After Epley—moderate to intense
	3	None	None
Right DHT	1	Persistent mild downbeat	None
	2	Left beat, persistent	None
	3	Right beat, ×30 s	None
Left DHT	1	Left torsional, then downbeat with right torsion (increase after return to sit)	Mild then intense during Epley (nose down position)
	2	Initial—right beat, persistent	Initial—moderate Repeat—intense, then mild
		Repeat—upbeating, left torsion ×15 s, then right beat	Repeat—intense, then mild
		After Epley—right beat	After Epley—none
	3	Initial/Repeat/first Epley—Upbeat, left torsion ×10 s, then downbeat, right torsion ×30 s	Initial/repeat/after first Epley—-mild
		After bow and yaw—upbeat, left torsion, reproduced in nose down position Epley	After bow and yaw—moderate
		After second Epley—none	None

All nystagmus instances observed were transient (<1 min duration) unless otherwise noted as persistent.

Moving from sit to supine induced a mild downbeat nystagmus. Right SRT showed a downbeat nystagmus without symptoms, while left SRT initially showed a right beat apogeotropic nystagmus less than 5 s that transitioned to a mild downbeat nystagmus with right torsion. Right DHT showed persistent downbeat nystagmus without clear torsion in either gaze position (i.e., eye in orbit); left DHT initially showed left torsional nystagmus less than 5 s that transitioned to downbeat with mild right torsion. Upon returning to sit from left DHT, he had a more pronounced downbeat and right torsional nystagmus. He was treated with Epley for left posterior semicircular canal BPPV, with increased downbeat with mild right torsion in the third position of the Epley and his most notable symptoms of vertigo. Following treatment, repeated right and left DHT showed minimal downbeat nystagmus and no symptoms, although upon returning to sit there was prolonged crescendo–decrescendo left torsional nystagmus with mild upbeat and vertigo. Further treatment on the initial visit was deferred due to time constraints.

On the return visit 6 days later, sit to supine with head flexed 30° showed a right beat nystagmus without symptoms. Right SRT showed a mild left beat (apogeotropic) nystagmus without symptoms, and left SRT showed a fast velocity right beat (apogeotropic) nystagmus with vertigo. After 28 s, the nystagmus transitioned to upbeating with left torsion with increased vertigo that lasted 17 s, then returned to a persistent horizontal right beating apogeotropic nystagmus with diminishing symptoms lasting more than a minute (Supplementary Video S3).

Right DHT showed left beating nystagmus that was persistent, without symptoms. Left DHT showed a right beat nystagmus with vertigo that was persistent. Upon return to sit, he had mild downbeat with left torsion that then transitioned to persistent left beating with mild vertigo. Right sidelying (patient's most symptomatic position at home) showed left beating, persistent nystagmus with mild vertigo. Bow showed a left beat nystagmus without symptoms, lean showed an upbeat left torsional nystagmus with vertigo lasting about 5 s that transitioned to right beating with vertigo.

Repeat left DHT showed upbeating left torsional nystagmus <15 s that transitioned to right beat horizontal nystagmus. He was treated with a left Epley CRM with reproduction of upbeating left torsional nystagmus and vertigo in the third position (right side lie), suggestive of treatment success (16). Following the maneuver, a repeat left DHT showed right beating nystagmus without vertigo. In left SRT, his right beat (apogeotropic) nystagmus intensified and remained persistent with associated vertigo. In right SRT, he had persistent left beat (apogeotropic) nystagmus without vertigo (mild nausea). He was then treated with a modified Gufoni for a right horizontal canal apogeotropic BPPV. There was mild left beat nystagmus in the initial right sidelying position, which intensified then went away after turning the nose toward the ceiling. There was no nystagmus during return to sit.

On his third visit, sit to supine showed a slow upbeat and left torsional nystagmus lasting less than 10 s. SRT to the right showed less than 10 s of slow geotropic nystagmus without vertigo; SRT to the left was negative. Return to sit showed a slow downbeating and right torsional nystagmus lasting less than 10 s. Right DHT showed a slow velocity right beating nystagmus that lasted 30 s without reversal on returning to sit. Left DHT showed an initial upbeating left torsional nystagmus lasting 10 s with vertigo, which transitioned to a slow downbeat nystagmus lasting 30–35 s. There was no reproduction of nystagmus or symptoms throughout an attempted Epley CRM for left posterior semicircular canal BPPV, and upon returning to sit, he had 10 s of downbeat nystagmus with right torsion.

Repeat left DHT showed an initial upbeating left torsional nystagmus lasting 10 s with vertigo, which transitioned to a slow downbeat nystagmus lasting 30–35 s, although this time there was a right torsional component. The downbeating nystagmus remained unchanged in a half-DHT (17). With return to sit, there was an increased velocity downbeat and right torsional nystagmus lasting about 10 s. A bow and yaw maneuver was attempted next given the persistent downbeating with right torsional nystagmus suggestive of a short-arm posterior canal BPPV, followed by a repeat left DHT that showed excitatory upbeat and left torsional nystagmus before transitioning to a transient downbeat. A second Epley CRM was completed with reproduction of excitatory nystagmus and vertigo in the third position, suggestive of treatment success (16). Repeat DHT and SRT were negative.

#### Case pearls and possible mechanisms

This case similarly highlights the importance of identifying canal-specific nystagmus patterns independent of the positional test being performed and the challenge of multi-canal BPPV. This was most notable on the second day of testing when the patient initially displayed right beating apogeotropic nystagmus before transiently changing to upbeat and left torsional nystagmus while in the left SRT. The bow and lean test helped lateralize the affected horizontal semicircular canal but was also beneficial in clearly revealing a concomitant left posterior canal BPPV (18).

In this challenging case, the patient had an initial downbeat nystagmus in both DHTs yet a right torsional component developed while in the left DHT that can only be generated from inhibition of the left posterior semicircular canal when BPPV is the culprit (note that excitation of the left anterior canal would cause downbeat with left torsion). As mentioned above, anterior canal BPPV is rare and distinguishing the torsional component in many cases may be difficult (9), although it must be advantaged by asking patients to change the eye in orbit position (3). In addition, Bhandari et al. (11) showed through three-dimensional simulations that upon return to sitting in an anterior canal BPPV, otoconia continues in an ampullofugal/excitatory direction, explaining the absence of nystagmus reversal after returning to sit. This patient had a clear reversal of nystagmus upon returning to sit that further indicated this was not likely to be from an anterior canal BPPV, which led the clinician to attempt an Epley maneuver for an affected left posterior semicircular canal.

Repeated testing within and between visits revealed the otoconia location would change following testing, treatment, or a return to daily activities. While it is rare, multi-canal BPPV occurs in ∼5.1% of cases (cross-sectional study of 3,975 patients with BPPV) (6). This patient responded to maneuvers for both the posterior and horizontal semicircular canals. With respect to the downbeat nystagmus observed after headshaking, Lee and Kim (19) showed 20% of patients with posterior semicircular canal BPPV can show a “perverted” vertical nystagmus. Yang et al. demonstrated that perverted post headshake nystagmus is not specific to central disorders (20).

## Discussion

BPPV represents a common and typically easy to treat cause of vertigo; however, there is a growing body of evidence demonstrating numerous variants that can make its diagnosis challenging. Understanding and leveraging the rules of vestibular physiology is critical to ensuring accurate and timely diagnosis and treatment. Removing visual fixation is especially critical in peripheral vestibular disorders such as BPPV. Özel et al. (21) demonstrated that positional nystagmus was suppressed in room light by as much as 66.1% when patients with BPPV were tested without blocking fixation. Asking patients to change eye in orbit (3) can further aid the diagnostic process. Video-Frenzel recording goggles allow for re-examination of positional nystagmus after testing that is helpful to ensure diagnostic accuracy and treatment success, and if needed, share with other clinicians.

## Data Availability

The original contributions presented in the study are included in the article/Supplementary Material; further inquiries can be directed to the corresponding author.
